# Reducing saturated fat intake lowers LDL-C but increases Lp(a) levels in African Americans: the GET-READI feeding trial

**DOI:** 10.1016/j.jlr.2023.100420

**Published:** 2023-07-22

**Authors:** Hayley G. Law, Muhammad A. Khan, Wei Zhang, Heejung Bang, Jennifer Rood, Marlene Most, Michael Lefevre, Lars Berglund, Byambaa Enkhmaa

**Affiliations:** 1 Department of Internal Medicine, School of Medicine, University of California Davis, Davis, CA, USA; 2 Department of Public Health Sciences, School of Medicine, University of California Davis, Davis, CA, USA; 3 Pennington Biomedical Research Center, Baton Rouge, LA, USA; 4 Department of Nutrition, Utah State University, Logan, UT, USA; 5 Center for Precision Medicine and Data Sciences, School of Medicine, University of California Davis, Davis, CA, USA

**Keywords:** Lipoprotein (a), Lipoprotein (a) metabolism, LDL, Dietary fat, Nutrition

## Abstract

Reducing dietary saturated fatty acids (SFA) intake results in a clinically significant lowering of low-density lipoprotein cholesterol (LDL-C) across ethnicities. In contrast, dietary SFA’s role in modulating emerging cardiovascular risk factors in different ethnicities remains poorly understood. Elevated levels of lipoprotein(a) [Lp(a)], an independent cardiovascular risk factor, disproportionally affect individuals of African descent. Here, we assessed the responses in Lp(a) levels to dietary SFA reduction in 166 African Americans enrolled in GET-READI (The Gene-Environment Trial on Response in African Americans to Dietary Intervention), a randomized controlled feeding trial. Participants were fed two diets in random order for 5 weeks each: *1*) an average American diet (AAD) (37% total fat: 16% SFA), and *2*) a diet similar to the Dietary Approaches to Stop Hypertension (DASH) diet (25% total fat: 6% SFA). The participants’ mean age was 35 years, 70% were women, the mean BMI was 28 kg/m^2^, and the mean LDL-C was 116 mg/dl. Compared to the AAD diet, LDL-C was reduced by the DASH-type diet (mean change: −12 mg/dl) as were total cholesterol (−16 mg/dl), HDL-C (−5 mg/dl), apoA-1 (−9 mg/dl) and apoB-100 (−5 mg/dl) (all *P* < 0.0001). In contrast, Lp(a) levels increased following the DASH-type diet compared with AAD (median: 58 vs. 44 mg/dl, *P* < 0.0001). In conclusion, in a large cohort of African Americans, reductions in SFA intake significantly increased Lp(a) levels while reducing LDL-C. Future studies are warranted to elucidate the mechanism(s) underlying the SFA reduction-induced increase in Lp(a) levels and its role in cardiovascular risk across populations.

Despite current advances in prevention and treatment strategies, cardiovascular disease (CVD) remains the leading cause of mortality in the U.S. Lifestyle modifications, including dietary changes, are among the first line of interventions to prevent and manage CVD risk (1, 2, 3). Dietary recommendations to reduce saturated fatty acids (SFA) intake result in a significant clinically meaningful lowering of low-density lipoprotein cholesterol (LDL-C). A meta-analysis of eight randomized controlled trials that lowered dietary SFA intake and replaced it with polyunsaturated fats (PUFA) reported a 19% risk reduction in coronary heart disease, corresponding to a 10% lower risk per 5% greater energy from PUFA (4).

Lipoprotein(a) [Lp(a)] is a particle with similarities to LDL-C but with a unique apolipoprotein(a) [apo(a)] component bound to apoB-100. Although the exact physiological functions of Lp(a) remain unknown, a large body of evidence supports its role as an independent, causal, and prevalent risk factor for CVD (5, 6). Lp(a) levels are, on average, 2- to 3-fold higher in African Americans than Caucasians, and the difference is not fully explained by genetic factors (7, 8). Available evidence suggests a role for other factors, including diet, in influencing Lp(a) levels (9). Indeed, dietary SFA reduction was associated with 7–20% increases in Lp(a) levels (10). However, limited data are available on African Americans. Notably, clinical measurements of LDL-C commonly include cholesterol carried on Lp(a) [Lp(a)-C]. Therefore, any SFA reduction-induced increase in Lp(a) levels could result in an underestimation of actual LDL-C lowering during dietary interventions. This effect could be particularly prominent in individuals or population groups with elevated Lp(a) levels, such as people of African descent. To address these evidence gaps, we conducted an in-depth analysis in The Gene-Environment Trial on Response in African Americans to Dietary Intervention (GET-READI) study, a randomized, controlled, and crossover feeding trial among 166 African Americans.

## Materials and methods

### Participants

The details of the recruitment and participants have been described previously (11). Briefly, the GET-READI trial recruited families of African descent with a minimum of two adult biological siblings in Baton Rouge, Louisiana, between 2001 and 2005. Families were selected based on the following: (1) at least two adult full siblings willing and eligible to participate in a 12-week dietary feeding trial and at least one biological parent available for DNA sampling or (2) at least three adult full siblings willing and eligible as noted above without regard to the availability of biological parents for DNA sampling. Additionally, at least one offspring was required to have an LDL-C above the 50^th^ percentile adjusted for age, race, and sex based on the National Health and Nutrition Examination Survey III data (12, 13). This percentile corresponded to an average LDL-C of 120 mg/dl for African American males and 117 mg/dl for African American females between 30 and 39 years. Overall, African American men and women aged 18–65 years, willing to only consume foods provided by the trial during the feeding periods, willing to abstain from the consumption of alcohol for 48 h prior to blood draw days, and willing to provide genetic material for testing were eligible to participate in the trial. The exclusion criteria applied to offspring participating in the dietary intervention included age (<18 and >45 years), LDL-C ≥200 mg/dl, triglycerides ≥500 mg/dl, or blood pressure ≥160 mmHg systolic or ≥95 mmHg diastolic. Additional exclusion criteria for the participating offspring included (1) lack of parent–offspring relationship verified through initial genetic testing; (2) documented presence of atherosclerotic disease; (3) diabetes mellitus; (4) antihypertensive medication; (5) renal, hepatic, endocrine, gastrointestinal or other systemic disease; (6) body mass index (BMI) ≥40; (7) for women: pregnancy, breastfeeding, or postpartum <6 months; (8) history of drug or alcohol abuse; (9) history of depression or mental illness requiring treatment or medication within the last 6 months; (10) multiple food allergies or significant food preferences or restrictions that would interfere with diet adherence; (11) chronic use of over-the-counter medication that would interfere with study endpoints including non-steroidal anti-inflammatory drugs, laxatives, and antacids; and (12) lifestyle or schedule incompatible with the study protocol. Participants were required to comply with dietary guidelines and consume at least one meal each weekday at the clinical center. The order of the diets was selected through a computer-based randomization process, and only study foods were permitted during the feeding phases of the trial. The participant recruitment process is shown in supplemental Fig. S1. The present report is based on the data of 166 individuals who completed both diet periods.

### Study design and controlled diets

Two well-controlled diets were provided to participants: one similar to the average American diet (AAD) and a diet similar to the Dietary Approaches to Stop Hypertension (DASH) diet, but with slightly lower levels of total fat (25% of energy intake) and saturated fat (6% of energy intake) (Table 1). The carbohydrates used to replace SFA in the DASH-type diet were primarily derived from fruits and vegetables, and the detailed regimen including types and servings generally mirrored those in previous studies using similar types of diets with ∼10% reduction in dietary SFA intake (11, 14, 16, 17). However, to enhance potassium and magnesium intake in the GET-READI study, fruits such as bananas and dark leafy greens made up a greater proportion of the participants’ diet than in the aforementioned studies. Sodium levels were designed to be held constant across diets at palatable levels as previous studies of the latter diet have documented blood pressure reduction in African Americans with modest hypertension, even in the absence of changes in sodium levels (14, 15). The trial included a 1-week run-in period to familiarize participants with the routine, followed by 5 weeks of one experimental diet, 1 week break, and 5 weeks of the other experimental diet. A 5-day menu cycle was used and the order of the two diets was randomized. All personnel involved in determining outcome variables were blinded with respect to the test diets. On weekdays, the participants consumed breakfast and dinner at the Pennington Biomedical Research Center (PBRC) dining facility. Each participant had their trays checked following the meals to ensure that all food was consumed. Weekday lunches and snacks were packaged for take-out and were distributed at breakfast. Weekend meals were packaged and distributed at the Friday dinner. Participants kept a daily diary recording adherence to study foods. Additionally, the levels of urinary minerals (potassium, magnesium, and sodium) were assessed for adherence. Overall dietary adherence was excellent according to the meal checks, daily diaries, and higher mean (SD) urinary potassium (65 ± 30 vs. 36 ± 14 mmol/L) and magnesium (9.4 ± 4.6 vs. 6.8 ± 3.5 mmol/L) levels following the DASH-type diet versus the AAD diet. In accordance with the diet design, the mean urinary sodium levels were similar at the end of the AAD versus at the end of the DASH-type diet (124 ± 53 vs. 119 ± 55 mmol/L). The study follows the Declaration of Helsinki principles, was approved by the institutional review board, and each participant provided written informed consent.

**Table 1:** Nutrient content and food group targets for experimental diets

Nutrients	AAD^b^	DASH-type diet^c^
Protein (% kcal)	15	18
Carbohydrate (% kcal)	48	57
Fat (% kcal)	37	25
Saturated fat (% kcal)	16	6
Monounsaturated fat (% kcal)	13	12
Polyunsaturated fat (% kcal)	8	7
Cholesterol (mg/d)^a^	300	150
Fiber (g/d)^a^	9	31
Potassium (mg/d)^a^	1,700	4,700
Magnesium (mg/d)^a^	165	500
Calcium (mg/d)^a^	450	1,240
Sodium (g/d)^a^	3	3

AAD, average American diet; DASH, Dietary Approaches to Stop Hypertension.

a Target levels for a 2,100 kcal diet.

b A diet similar to that consumed by Americans, average American diet (AAD), but with slightly higher total fat and saturated fat, and slightly lower fiber, magnesium, potassium, and calcium.

c A diet similar to the DASH Combination Diet (Step 2 DASH) but with slightly lower levels of total fat and saturated fat. The modifications to the fat and fatty acid levels of the diets were designed to maximize the ability to detect gene-diet interactions for LDL-C while still endeavoring to keep the diets relevant. Collectively, the contrasts in the fats, protein, fiber, potassium, magnesium, and calcium have been documented to produce changes in blood pressure in African Americans with high normal blood pressure, even in the absence of changes in sodium levels (14, 15). Sodium levels were designed to be held constant across diets at palatable levels. Attempts to impose significant sodium restrictions on the DASH-type diet would present challenges with compliance and would provide difficulty in diet preparation without substantial additional benefit in terms of blood pressure reduction.

### Study endpoints, data collection, and laboratory analyses

The primary endpoints were LDL-C and systolic and diastolic blood pressures, with additional secondary endpoints that included triglycerides, high-density lipoprotein cholesterol (HDL-C), and Lp(a). Fasting blood samples for key lipid and lipoprotein endpoints were collected in duplicate following the diet run-in period and in triplicate during the final fifth week of each diet period and were stored at −80°C. The averages of replicate measurements for the endpoints, including Lp(a), taken at the end of the AAD diet or the DASH-type diet were used for analyses. This approach allowed a new steady-state for Lp(a) as previously shown (16). Thus, a total of 6 weeks between the compared time points minimizes confounding by the randomization order. Clinical laboratory variables, including cholesterol and lipoproteins, were analyzed using a Beckman array system, and Lp(a) concentration was measured with a rate nephelometry assay at the PBRC clinical chemistry laboratory.

### Statistical analyses

We summarized participant characteristics and outcomes data with standard descriptive statistics using mean and standard deviation (SD) and median and interquartile range (IQR) for continuous variables (after examining the skewness of data) and frequency (%) for categorical variables. Data analyses were performed for all participants using the participant level data first, then by subgroups of sex or high versus low Lp(a) levels. The cutoff point separating high and low Lp(a) was defined as 50 mg/dl based on recent guidelines that identified it as a risk-enhancing factor in the development of CVD (2, 3, 6). Due to the exploratory nature of the latter analysis and acknowledging that Lp(a) can be affected by the study intervention, *P*-values were not calculated. We computed mean absolute and percent changes for each clinical and laboratory variable. When we compared non-independent data between the two diet groups, due to the crossover nature of the study design, we computed statistical significance accounting for dependency via paired *t* test. Additionally, we conducted the analyses in mixed effect models to account for repeated measures and clustering by participant, and then further by sibling (18). For an ancillary analysis, we fitted linear regression for the changes in Lp(a) and changes in other laboratory variables, with and without adjustments for age and sex. Statistical significance is reported with unadjusted values for multiple testing. SAS version 9.4 was used for data analyses.

## Results

### Participant characteristics

The baseline demographic characteristics of the study participants have been described previously (11). The mean age of participants was 35 years (range: 18–60 years). The majority of the participants were women (∼70%). The prevalence of a smoking history or alcohol consumption was 7% and 3%, respectively. The mean BMI was 28 ± 5 kg/m^2^, while the mean systolic and diastolic blood pressure were 118 ± 10 mmHg (range: 89–159) and 75 ± 7 mmHg (range: 55–100), respectively. Characteristics of all participants who completed both dietary periods are shown in Table 2. Compared to men, women participants had higher levels of BMI, body fat, total cholesterol, HDL-C, and apoA-1 but lower triglyceride levels (Table 2).

**Table 2:** Characteristics of the study participants

Variables	All (n = 166)	Women (n = 116)	Men (n = 50)
Age (years)	35 ± 11	37 ± 11	31 ± 10
Body weight (kg)	80 ± 15	78 ± 16	83 ± 14
Body mass index (kg/m^2^)	28 ± 5	29 ± 6	26 ± 4
Body fat (% of body weight)	31 ± 10	36 ± 6	27 ± 10
Diastolic blood pressure (mm Hg)	75 ± 7	76 ± 7	74 ± 7
Systolic blood pressure (mm Hg)	118 ± 10	116 ± 10	121 ± 9
Total cholesterol (mg/dl)	186 ± 32	191 ± 32	176 ± 31
LDL cholesterol (mg/dl)	116 ± 29	118 ± 30	111 ± 26
HDL cholesterol (mg/dl)	55 ± 15	58 ± 15	48 ± 10
Triglycerides (mg/dl)	71 (51–90)	65 (50–88)	82 (54–94)
Apolipoprotein A-1 (mg/dl)	136 ± 32	140 ± 33	126 ± 25
Apolipoprotein B-100 (mg/dl)	91 ± 21	92 ± 21	90 ± 22
Glucose (mg/dl)	94 ± 11	94 ± 12	94 ± 9
Insulin (uU/ml)	12 ± 7	13 ± 7	10 ± 6

Data are shown as mean ± SD, excluding triglycerides shown as median (25^th^–75^th^ percentiles).

### Clinical and laboratory values at the end of the AAD diet and DASH-type diet, respectively, and changes (unit and percent) from AAD to DASH-type diet

Clinical and laboratory values at the end of the two dietary periods in all participants are shown in Table 3. In all participants, both systolic (mean: 115 vs. 117 mmHg) and diastolic (74 vs. 75 mmHg) blood pressure and concentrations of total cholesterol (169 vs. 186 mg/dl), apoB-100 (85 vs. 90 mg/dl), HDL-C (50 vs. 56 mg/dl), and apoA-1 (127 vs. 136 mg/dl) were lower following the DASH-type diet compared with the AAD diet. Expectedly, LDL-C concentrations were significantly lower at the end of the DASH-type diet compared with the end of the AAD diet (103 vs. 115 mg/dl). In contrast, Lp(a) concentrations were elevated with the DASH-type diet compared to the AAD diet (median: 58 vs. 44 mg/dl) as were triglyceride levels (median: 70 vs. 64 mg/dl). Notably, the prevalence of high Lp(a) (≥50 mg/dl) increased from 43% with the AAD diet to 53% with the DASH-type diet. Overall, the results for men and women mirrored those for the entire cohort (supplemental Table S1).

**Table 3:** Clinical and laboratory values at the end of the AAD diet and DASH-type diet, respectively, and changes (unit and percent) from AAD to DASH-type diet in all participants

Variables	AAD	DASH-type	Unit change^a^	% Change	*P*-value^b^
Body weight (kg)	80 ± 16	79 ± 15	−0.4 ± 1	−0.5 ± 2	0.0002/0.010/0.009
Diastolic blood pressure (mmHg)	75 ± 7	74 ± 7	−1.2 ± 4	−1.4 ± 5	0.0003/0.004/0.003
Systolic blood pressure (mmHg)	117 ± 10	115 ± 9	−2.3 ± 6	−1.7 ± 5	<0.0001/<0.0001/0.006
Total cholesterol (mg/dl)	186 ± 32	169 ± 31	−16 ± 19	−8 ± 9	<0.0001
LDL cholesterol (mg/dl)	115 ± 28	103 ± 26	−12 ± 15	−10 ± 12	<0.0001
HDL cholesterol (mg/dl)	56 ± 15	50 ± 13	−5 ± 6	−8 ± 10	<0.0001
Triglycerides (mg/dl)	64 (49–85)	70 (51–95)	4 ± 22	8 ± 26	0.026/0.090/0.088
Lipoprotein(a) (mg/dl)	44 (22–80)	58 (28–94)	11 ± 11	24 ± 25	<0.0001
Apolipoprotein A-1 (mg/dl)	136 ± 31	127 ± 29	−9 ± 11	−6 ± 8	<0.0001
Apolipoprotein B-100 (mg/dl)	90 ± 20	85 ± 20	−5 ± 10	−6 ± 10	<0.0001
Glucose (mg/dl)	93 ± 11	92 ± 10	−0.9 ± 5	−0.7 ± 5	0.027/0.045/0.045
Insulin (μU/ml)	12 ± 7	12 ± 7	−0.2 ± 4	5 ± 44	0.559/0.731/0.750

Data at the end of each diet period (AAD and DASH-type) are shown as mean ± SD with the exception of triglycerides and Lp(a) levels which are shown as median (25^th^–75^th^ percentiles). Data for changes (unit and percent) are shown as mean ± SD for all variables. Unit and percent changes were calculated using the data collected at the end (week five) of each diet period.

AAD, average American diet; DASH, Dietary Approaches to Stop Hypertension.

a Unit changes are in kg, mmHg, and μU/ml for body weight, blood pressure, and insulin, respectively. For all cholesterol and apolipoprotein values, unit changes are shown in mg/dl.

b *P*-values are for significance for absolute and percent changes. Three *P*-values from paired *t* test, mixed effect model with 1 random effect (subject), and mixed effect model with 2 random effects (subject and siblings). For mixed effect models, *P*-value is for treatment and period interaction. When period effect is not statistically significant, *P*-value for the treatment term is reported. If the three *P*-values were identical, a single value was reported. Since Lp(a) levels were not assessed at baseline, two repeated measures were included in the mixed effect model for longitudinal data analysis; for all other variables, three measurements were included.

**Fig. 1:**
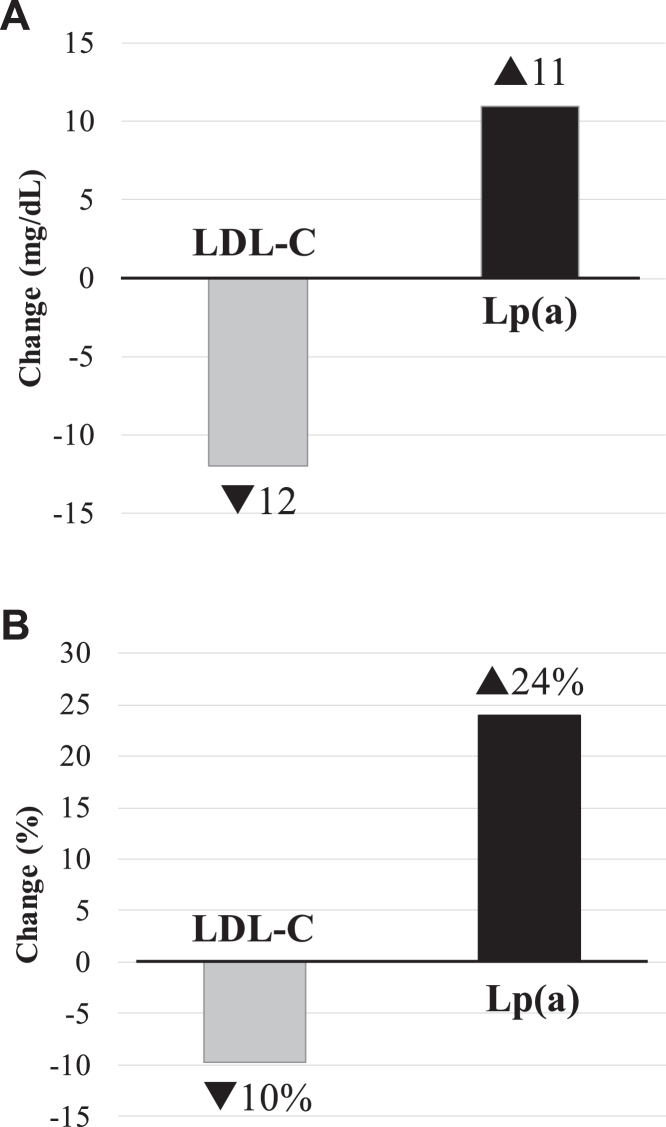
Opposite effects of dietary saturated fat reduction on LDL-C versus Lp(a) concentrations in all participants. Data are shown for mean changes as mg/dl (A) and percent (%) (B) for LDL and Lp(a) concentrations from the AAD diet to the DASH-type diet in all participants.

Furthermore, in all participants, the degree of reductions from the AAD to the DASH-type diet was significant for body weight (mean: −0.4 kg; −0.5%, *P* = 0.0002), systolic blood pressure (−2.3 mmHg; −1.7%, *P* < 0.0001), and diastolic blood pressure (−1.2 mmHg; −1.4%, *P* = 0.0003) (Table 3). Similarly, the differences from the AAD to the DASH-type diet in total cholesterol (mean: −16 mg/dl; −8%), HDL-C (−5 mg/dl; −8%), apoB-100 (−5 mg/dl; −6%), and apoA-1 (−9 mg/dl; −6%) (*P* < 0.0001 for all) as well as in glucose (−0.9 mg/dl; −0.7%, *P* = 0.027) were significant for all participants. Notably, the average LDL-C decrease by 12 mg/dl in all participants corresponded to a 10% reduction (*P* < 0.0001). In contrast to these findings, Lp(a) concentration increased from the AAD to the DASH-type diet, on average, by 11 mg/dl corresponding to a 24% increase (*P* < 0.0001). The opposite responses in LDL-C and Lp(a) concentrations to the DASH-type diet are illustrated in Fig. 1. In addition, there was a small but significant increase in the triglyceride concentration from the AAD diet to the DASH-type diet in all participants (4 mg/dl; 8%, *P* = 0.026). When adjusted for diet order and sibling relationship, these changes remained significant, excluding only the change in triglycerides (Table 3). Overall, the findings among women participants mirrored those for the entire cohort. However, among men, differences between the AAD and the DASH-type diets did not reach significance for body weight, diastolic blood pressure, and triglyceride concentrations (supplemental Table S1).

### Associations between changes in Lp(a) levels and changes in other clinical and laboratory variables from the AAD to the DASH-type diet in all participants

In all participants, changes in Lp(a) levels were significantly and negatively associated with changes in HDL-C and apoA-1 concentrations before and after adjustments for age and sex (HDL-C: before β = −0.0067, *P* = 0.012; after β = −0.0069, *P* = 0.011; apoA-1: before β = −0.0049, *P* = 0.0004; after β = −0.0049, *P* = 0.001) (supplemental Table S2).

### The effect of the DASH-type diet intervention by high and low Lp(a) groups in all participants

Next, we explored the effect of the DASH-type diet intervention for high and low Lp(a) groups using a cut-off Lp(a) level of 50 mg/dl at the end of the AAD diet period. The high Lp(a) group included 72 participants (43%) with Lp(a) levels ≥50 mg/dl (supplemental Table S3). The mean change in Lp(a) levels in the high Lp(a) group was substantially greater than that of the low Lp(a) group (16 vs. 7.5 mg/dl), while the extent of reductions in LDL-C was similar between the high and the low Lp(a) groups. Other changes in laboratory and clinical variables were not notably different between the high and low Lp(a) groups (supplemental Table S3).

## Discussion

In GET-READI, a randomized controlled cross-over feeding trial among 166 African Americans, a reduction in dietary saturated fat intake from 16% to 6%, replaced primarily with carbohydrates, resulted in significant reductions in systolic and diastolic blood pressure and LDL-C concentrations. In contrast, a reduction in dietary saturated fat intake led to a significant 24% increase in Lp(a) concentrations. Therefore, the replacement of dietary saturated fats with carbohydrates generated a discordant response in two highly atherogenic lipoproteins in African Americans. Furthermore, these opposing changes in LDL-C and Lp(a) concentrations were accompanied by small but significant changes in HDL-C (decrease) and triglyceride (increase) concentrations.

Plasma Lp(a) levels are mostly genetically determined (19). The study of non-genetic determinants of Lp(a) levels, therefore, has been a challenging task that requires consideration of adequate sample sizes and prospective study designs (9). Most available evidence from well-designed dietary studies suggests that replacement of saturated fats with other macronutrients—such as monounsaturated fat (MUFA) or carbohydrates—increases Lp(a) levels, while consistently decreasing LDL-C (9, 10, 20). This diet-mediated effect is one of the few non-genetic factors to influence Lp(a) levels and does not align with the well-known atherogenic and cholesterol-raising properties of SFA. The effects of reducing dietary SFA and replacing it with either carbohydrates or MUFA on CVD risk factors have been investigated in detail in two multicenter randomized controlled crossover Dietary Effects on Lipoproteins and Thrombogenic Activity (DELTA) trials (16, 21, 22). The DELTA 1 trial evaluated the efficacy of the American Heart Association Step 1 diet (7% energy from SFA replaced with carbohydrates) and a diet with further reduction in SFA (a low-SFA diet: 11% energy from SFA replaced with carbohydrates) to improve CVD risk factors in a healthy normolipidemic cohort which included 26 (25%) African Americans (16, 22). The DELTA 2 study evaluated whether carbohydrates (Step 1 diet) or MUFA was the preferred replacement for SFA (each replacing 7% energy) in a cohort of metabolically at-risk adults that included 10 (12%) African Americans (21). In the two DELTA studies, the replacement of SFA with carbohydrates or MUFA resulted in significant reductions in LDL-C (7–11%). In line with these reports, we observed a 10% (−12 mg/dl) reduction in LDL-C when SFA was replaced primarily with carbohydrates. This extent of LDL-C lowering can translate to a 10% relative risk reduction for major CVD events based on therapeutic studies (23). In contrast, Lp(a) level increased ∼15% between the AAD and the low-SFA diets in DELTA 1 (16). In DELTA 2, Lp(a) increased with both the Step 1 (20%) and MUFA (11%) diets compared with the AAD (21). In the present study of 166 African Americans, we observed a larger increase (24%) in Lp(a) levels from the AAD diet to the DASH-type diet. This extent of increase in Lp(a) is expected to generate a 7% increase in heart disease outcomes based on genetic studies (24). This larger increase in Lp(a) levels, when compared with previous studies, indicates that Lp(a) level changes may be of particular importance to CVD risk in those with high levels of Lp(a).

**Fig. 2:**
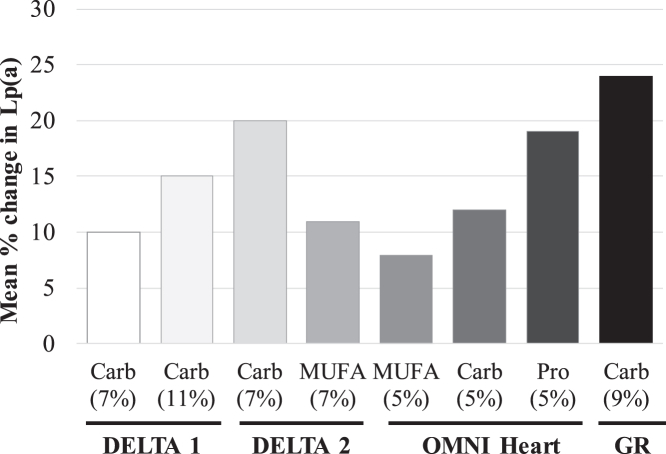
Effect of replacement of dietary saturated fats with other nutrients on Lp(a) concentrations in randomized controlled metabolic feeding trials. Data are shown as mean percent (%) change in Lp(a) concentration from the average American diet (DELTA 1, DELTA 2 and GET-READI) or the baseline habitual diet (OMNI Heart) to the respective healthy diets that replaced saturated fats with another macronutrient. Percentages under macronutrient names indicate the % replacement of SFA with the corresponding nutrient. Data are from all participants in the DELTA 1 (n = 103, including 26 African Americans) (16) and DELTA 2 (n = 85, including 10 African Americans) (21) trials, African American participants only in the OMNI Heart trial (n = 89) (25), and all participants in the GET-READI (n = 166 African Americans). Data were obtained from Table 3 in the cited references corresponding to each study (16, 21, 25). Carb, carbohydrate; DELTA, Dietary Effects on Lipoproteins and Thrombogenic Activity; GR, GET-READI (The Gene-Environment Trial on Response in African Americans to Dietary Intervention); MUFA, monounsaturated fat; OMNI, Optimal Macronutrient Intake Trial to Prevent Heart Disease; Pro, protein.

The Optimal Macronutrient Intake Trial to Prevent Heart Disease (OMNI Heart) controlled dietary trial was one of the largest studies investigating racial/ethnic differences in Lp(a) response to dietary changes (25). The trial enrolled 89 African American and 66 White participants. In this study, Lp(a) changes were greater in African American versus White participants across the three controlled diets that replaced SFA with either carbohydrates, protein, or unsaturated fat compared with the participants’ habitual baseline diet (25). These findings suggested that there might be an inherent racial difference in Lp(a) metabolism depending on macronutrient composition. Among the OMNI Heart African American participants, Lp(a) rose by ∼4 mg/dl from the baseline mean level of 32 mg/dl during the carbohydrate-rich diet (58% of energy from carbohydrate, 27% total fat, including 6% SFA, and 15% protein). This extent of increase in Lp(a) level is lower than what was observed in the current study (11 mg/dl). This heterogeneous response in Lp(a) level could be, in part, due to differences in the study diets and cohort characteristics between the two trials. Compared with the OMNI Heart African American participants, the GET-READI study participants were younger (mean age: 35 vs. 52 years) and had a considerably higher mean Lp(a) level (54 vs. 32 mg/dl). The overall macronutrient compositions of the high-carbohydrate diet in the OMNI Heart trial and the GET-READI study diet were similar, with a slight difference in the protein energy, which was greater in GET-READI versus the OMNI Heart trial (18% vs. 15%). Interestingly, the OMNI Heart trial showed that the largest increase in Lp(a) level was observed when participants consumed the protein-rich diet, followed by the carbohydrate-rich and MUFA-rich diets (25). It is tempting to speculate that the higher protein energy in the GET-READI study diet versus the OMNI Heart carbohydrate-rich diet may have contributed to the larger increase in Lp(a) levels in the current study. In addition, compared with the AAD diet, the DASH-type diet replaced ∼10% energy from SFA with carbohydrates, while the corresponding energy replacement with carbohydrates in the OMNI Heart trial was ∼5%. Taken together, the findings in this and previous studies suggest a specific effect of SFA reduction on Lp(a), which might impact strategies for dietary guidance. Thus, substitutions with dietary unsaturated fats may be a preferable regimen over protein- or carbohydrate-based regimens with regards to Lp(a). Furthermore, we did not observe sex-specific differences in the effect of diet on Lp(a) levels, in line with other reports (16). In Fig. 2, we illustrate the current study findings (100% African Americans) in relation to those in the DELTA 1 (25% African American participants) (16), DELTA 2 (12% African American participants) (21), and the OMNI Heart (57% African American participants) (25) trials.

In the DELTA (16, 21, 22) and the OMNI Heart (25) trials, participants consumed each diet for 6–8 weeks. These trials were of adequate duration to detect changes in LDL-C and Lp(a) levels and, notably, means and variance became stable from week 5 of each diet period in the two DELTA studies (16, 21, 22). These results justify the 5-week feeding period in the GET-READI study. Further, most clinical measurements of LDL-C include the cholesterol contained in Lp(a). This can lead to an inaccurate estimation of the actual LDL-C response to interventions in the presence of an elevated Lp(a) level. Therefore, precision CVD risk assessment and management will likely require measurement of LDL-C that is not confounded by Lp(a) content or a direct measurement of Lp(a)-C. Such precision-guided assessments will be important to accurately quantify the actual reduction in LDL-C in the context of increased Lp(a) levels during dietary interventions.

In individuals with low Lp(a) levels, a reduction in LDL-C remains an important goal for risk reduction. However, in individuals with very high Lp(a) levels and minor-to-modestly elevated LDL-C levels, an increase in Lp(a) level due to SFA reduction could become an important risk determinant. Under such conditions, more informed and individualized dietary advice would be necessary. Given the high prevalence of elevated Lp(a) (∼20–25% of the population, i.e., ∼1.4 B people worldwide) (26), these findings highlight the importance of precision nutrition approaches that move beyond a “one-size-fits-all” dietary prescription for optimal health and disease prevention (27). In support of this, a recent perspective discussed the contrasting effects of replacing dietary SFA on Lp(a) and LDL-C—the two highly atherogenic lipoproteins—in further depth and highlighted the importance of considering their relative balance during dietary interventions to manage cardiovascular risk (28). For every 39 mg/dl (1 mmol/L) increase, the observational hazard ratio was 1.6 for Lp(a) and 1.3 for LDL-C, and the causal risk ratio in corresponding genetic analyses was 2.0 for Lp(a) and 2.1 for LDL-C (29). This type of comparison supports the use of the entire range of Lp(a) and LDL-C to better understand the relative risk for both LDL-C and Lp(a) (30). Overall, the clinical importance of the increase in Lp(a) during dietary SFA reduction may be underappreciated despite its potential relevance in managing CVD risk in those with high Lp(a) levels.

A study by Faghihnia *et al.* (31) found a significant 2.2 mg/dl mean increase in Lp(a) levels following an 8% reduction in SFA replaced with carbohydrates in healthy participants. This response is substantially lower than what was observed in the present study. However, the cohort in Faghihnia *et al.*’s study was primarily (97%) male, in contrast to the majority female cohort (∼70%) in the present study, and the race of Faghihnia *et al.*’s participants was not reported. Despite these differences, it is noteworthy that the Lp(a) results of the Faghihnia *et al.* and the present studies are congruous in terms of the direction of effect. Although we observed significant associations of changes in Lp(a) with changes in HDL-C and ApoA-1, Faghihnia *et al.* (31) reported no significant associations of diet-induced changes in these variables with Lp(a). Overall, differences in the macronutrient composition of the test diets, Lp(a) levels, and overall participant characteristics between this and Faghihnia *et al.*’s (31) study likely contribute to these inconsistent observations. The negative associations of changes in Lp(a) with changes in HDL-C and ApoA-1 in our study might point towards a specific effect of saturated fats.

The study has some strengths and limitations. We studied a large number of African American men and women recruited in a single geographical location. In the U.S., and particularly in the South, African American individuals disproportionately suffer from CVD and stroke, warranting detailed investigations into risk factors and their responses to treatment modalities. The present study contributes to addressing this issue. The diets tested are based on common food-based eating patterns and are representative of the current guidelines for CVD prevention and management. The findings, therefore, are relevant to the free-living U.S. population. Notably, nearly half of the meals during the entire study period were consumed on-site at the PBRC, which has extensive experience conducting such studies (32). Furthermore, the GET-READI trial was of adequate duration to detect changes in both LDL-C and Lp(a) levels; however, studies with extended durations are required to fully delineate the long-term effect on CVD outcomes by dietary SFA reduction-induced increases in Lp(a) levels. Another limitation may be the fewer number of recruited men, which may explain the lack of statistically significant changes in men for some variables. Although we found no indication that our analyses were confounded by sex differences, we acknowledge the need for future studies with more men, or ones properly targeted for sex differences, to further clarify this issue. Furthermore, calibrators of nephelometric assays are usually selected to have high Lp(a) levels (33) with a potential consequence of overestimating Lp(a) values for cohorts with low Lp(a) levels (e.g., all White participants). However, in this African American cohort with generally higher Lp(a) levels, this bias would be minimized. The analyses by high and low Lp(a) levels were exploratory in nature because this study was not statistically powered to address this issue, and due to the fact that Lp(a) was affected by the study interventions. Finally, more studies are needed to elucidate the role of genetic factors, including apo(a) size polymorphism, in Lp(a) response to dietary changes and associated health outcomes. Nonetheless, the findings in the present study contribute to a better understanding of the regulation of Lp(a) levels based on a well-designed controlled dietary study in a large number of African Americans.

In conclusion, controlled feeding of a healthy DASH-type diet with a lower SFA and a higher carbohydrate content increased Lp(a) levels by 24% (when compared with the AAD diet) in African Americans. This atherogenic response in Lp(a) levels to dietary SFA reduction is in sharp contrast to the well-known LDL-C lowering effect of dietary SFA reduction. The findings suggest that dietary SFA reduction exerts an opposing effect on the circulating levels of two highly-atherogenic lipoproteins. Future studies are warranted to elucidate the mechanism(s) underlying the SFA reduction-induced increase in Lp(a) levels, responses in Lp(a) atherogenic properties (e.g., cholesterol and/or oxidized phospholipids) to dietary changes, and their roles in cardiovascular outcomes across population groups.

## Data availability

The datasets generated and analyzed during the present study are available from the corresponding author Enkhmaa Byambaa (ebyambaa@ucdavis.edu) on request.

## Supplemental data

This article contains supplemental data.

## Conflict of interest

The authors declare that they have no known competing financial interests or personal relationships that could have appeared to influence the work reported in this paper.
