# Supplementary material for: Reducing saturated fat intake lowers LDL-C but increases Lp(a) levels in African Americans: the GET-READI feeding trial
Source: J Lipid Res. 2023 Jul 22;64(9):100420. doi: 10.1016/j.jlr.2023.100420 (PMC10445453; doi:10.1016/j.jlr.2023.100420)
Supplement: Supplemental Figure S1 and Tables S1–S3 [file mmc1.docx]

**SUPPLEMENTAL MATERIALS**

**Reducing Saturated Fat Intake Lowers LDL-C but Increases Lp(a) levels in African Americans: The GET-READI Feeding Trial**

Hayley G. Law^a^, Muhammad A. Khan^a^, Wei Zhang^a^, Heejung Bang^b^, Jennifer Rood^d^, Marlene Most^d^, Michael Lefevre^d, e^, Lars Berglund^a^ and Byambaa Enkhmaa^a, c,*^

Departments of ^a^Internal Medicine and ^b^Public Health Sciences, and ^c^Center for Precision Medicine and Data Sciences, School of Medicine, University of California Davis, Davis, CA, USA; ^d^Pennington Biomedical Research Center, Baton Rouge, LA, USA; ^e^Department of Nutrition, Utah State University, Utah, USA.

**Supplemental Fig. S1. CONSORT chart**

**Reasons for loss at screening (n=1152):**

Lack of interest: 43%

Sibling/parent did not meet eligibility criteria: 19%

Patient did not meet eligibility criteria: 15%

Medical exclusion: 6%

Logistics: 5%

Unable/unwilling to eat study diet: 4%

Other: 8%

Included in phone screening

n=1483

Randomized

n=264

Completed both diet periods and included in data analyses

n=166

**Reasons for loss (n=57):**

One or more siblings did not want to proceed

Diet and/or logistical issues

**Reasons for loss (n=40):**

Patient or sibling(s) did not complete all diet periods

Completed Run-In

n=206

Completed screening

n=266

**Reasons for loss (n=2):**

Failure to show up

**Supplemental Table S1.** **Clinical and laboratory variables at the end of the AAD diet and DASH-type diet, respectively, and changes (unit and percent) from AAD to the DASH-type diets in women and men separately**

| **Variables** | **Women** | | | | | **Men** | | | | |
| --- | --- | --- | --- | --- | --- | --- | --- | --- | --- | --- |
|  | *AAD* | *DASH-type* | *Unit change^a^* | *%  change* | *P-*  *value^b^* | *AAD* | *DASH-type* | *Unit change^a^* | *%  change* | *P-*  *value^b^* |
| Body weight | 79 ± 16 | 78 ± 16 | -0.4 ± 1 | -0.5 ± 2 | 0.001 | 83 ± 14 | 83 ± 14 | -0.4 ± 2 | -0.4 ± 2 | 0.060 |
| Diastolic BP | 76 **± 7** | 74 **± 7** | -1.3 ± 5 | -1.6 ± 6 | 0.002 | 73 ± 7 | 72 ± 7 | -0.8 ± 3 | -1 ± 4 | 0.070 |
| Systolic BP | 116 ± 11 | 113 **±** 9 | -2.6 ± 6 | -2 ± 5 | <0.0001 | 120 ± 7 | 119 ± 7 | -1.5 ± 5 | -1.2 ± 4 | 0.043 |
| Total cholesterol | 190 ± 33 | 174 ± 28 | -16 ± 18 | -8 ± 8 | <0.0001 | 175 ± 29 | 160 ± 32 | -17 ± 21 | -9 ± 10 | <0.0001 |
| LDL cholesterol | 117 ± 29 | 106 ± 25 | -11 ± 16 | -9 ± 12 | <0.0001 | 110 ± 23 | 97 ± 26 | -13 ± 17 | -12 ± 13 | <0.0001 |
| HDL cholesterol | 58 ± 16 | 53 ± 14 | -5 ± 6 | -9 ± 10 | <0.0001 | 49 ± 11 | 45 ± 10 | -4 ± 5 | -8 ± 10 | <0.0001 |
| Triglycerides | 60  (50–83) | 69  (51–96) | 4 ± 20 | 8 ± 21 | 0.022 | 69  (49–91) | 70  (52–93) | 3 ± 27 | 8 ± 37 | 0.445 |
| Lipoprotein(a) | 47  (25–84) | 59  (34–99) | 11 ± 11 | 23 ± 22 | <0.0001 | 36  (18–61) | 52  (18–82) | 11 ± 12 | 26 ± 31 | <0.0001 |
| ApoA-1 | 139 ± 33 | 131 ± 31 | -9 ± 12 | -6 ± 8 | <0.0001 | 127 ± 24 | 118 ± 22 | -9 ± 9 | -7 ± 7 | <0.0001 |
| ApoB-100 | 91 ± 21 | 87 ± 19 | -5 ± 9 | -5 ± 10 | <0.0001 | 87 ± 18 | 81 ± 21 | -7 ± 11 | -8 ± 12 | <0.0001 |
| Glucose | 93 ± 11 | 92 ± 10 | -0.6 ± 4 | -0.5 ± 5 | 0.122 | 93 ± 11 | 92 ± 9 | -1.5 ± 7 | -1.2 ± 7 | 0.119 |
| Insulin | 12 ± 7 | 12 ± 6 | -0.3 ± 3 | 0.6 ± 25 | 0.334 | 10 ± 7 | 10 ± 7 | 0.1 ± 5 | 16 ± 71 | 0.915 |

Data at the end of each diet period (AAD and DASH-type) are shown as mean ± SD with the exception of triglycerides and Lp(a) levels which are shown as median (25^th^–75^th^ percentiles). Data for changes (unit and percent) are shown as mean ± SD for all variables.

*^a^*: Unit changes are in kg, mmHg, and uU/mL for body weight, blood pressure, and insulin, respectively. For all cholesterol and apolipoprotein values, unit changes are shown in mg/dL.

*^b^*: *P*-values represent significance for both absolute and percent changes.

*Abbreviations*: AAD, average American diet; Apo, apolipoprotein; BP, blood pressure; DASH, Dietary Approaches to Stop Hypertension;

**Supplemental Table S2. Regression analyses for the associations of changes in Lp(a) with changes in other laboratory variables in all participants before and after adjustments for age and sex**

| **Variables** | **Before adjustment** | | **After adjustment** | |
| --- | --- | --- | --- | --- |
|  | *β* | *P-value* | *β* | *P-value* |
| Body weight | -0.0187 | 0.091 | -0.0186 | 0.094 |
| Diastolic blood pressure | -0.0001 | 0.980 | -0.0002 | 0.950 |
| Systolic blood pressure | -0.0010 | 0.697 | -0.0012 | 0.649 |
| Total cholesterol | -0.0006 | 0.499 | -0.0005 | 0.511 |
| LDL cholesterol | 0.0007 | 0.522 | 0.0007 | 0.495 |
| HDL cholesterol | -0.0067 | 0.012 | -0.0069 | 0.011 |
| Triglycerides (log) | -0.0595 | 0.379 | -0.0599 | 0.383 |
| Apolipoprotein A-1 | -0.0049 | 0.0004 | -0.0049 | 0.001 |
| Apolipoprotein B-100 | 0.0013 | 0.436 | 0.0014 | 0.405 |
| Glucose | -0.0022 | 0.468 | -0.0021 | 0.482 |
| Insulin (log) | -0.0783 | 0.095 | -0.0799 | 0.091 |

**Supplemental Table S3. Effect of intervention on clinical and laboratory variables by high and low Lp(a) groups in all participants**

| **Variables** | **Lp(a) level** | | | |
| --- | --- | --- | --- | --- |
|  | **≥50 mg/dL (n=72)** | | **<50 mg/dL (n=94)** | |
|  | *unit change^a^* | *% change* | *unit change^a^* | *% change* |
| Body weight | -0.4 ± 0.17 | -0.5 ± 0.00 | -0.4 ± 0.14 | -0.4 ± 0.00 |
| Diastolic blood pressure | -1.2 ± 0.48 | -1.5 ± 0.01 | -1.1 ± 0.44 | -1.3 ± 0.01 |
| Systolic blood pressure | -2.4 ± 0.76 | -1.9 ± 0.01 | -2.2 ± 0.59 | -1.7 ± 0.01 |
| Total cholesterol | -17 ± 2.07 | -8.7 ± 0.01 | -15 ± 2.03 | -8.2 ± 0.01 |
| LDL cholesterol | -13 ± 1.63 | -11 ± 0.01 | -11 ± 1.72 | -9.2 ± 0.01 |
| HDL cholesterol | -5.0 ± 0.75 | -7.8 ± 0.01 | -5.0 ± 0.56 | -8.8 ± 0.01 |
| Lipoprotein(a) | 16 ± 1.51 | 19 ± 0.02 | 7.5 ± 0.84 | 27 ± 0.03 |
| Triglycerides | 3.6 ± 1.78 | 8.4 ± 0.02 | 4.0 ± 2.70 | 8.3 ± 0.03 |
| Apolipoprotein B-100 | -5.8 ± 0.99 | -6.4 ± 0.01 | -5.2 ± 1.09 | -5.3 ± 0.01 |
| Apolipoprotein A-1 | -9.0 ± 1.31 | -6.1 ± 0.01 | -8.5 ± 1.15 | -6.2 ± 0.01 |
| Glucose | -1.0 ± 0.46 | -1.0 ± 0.01 | -0.8 ± 0.62 | -0.6 ± 0.01 |
| Insulin | -0.4 ± 0.50 | 4.5 ± 0.10 | 0.01 ± 0.41 | 5.7 ± 0.04 |

Data are shown as mean ± standard error of the mean.

*^a^*: Unit changes are in kg, mmHg, and uU/mL for body weight, blood pressure, and insulin, respectively. For all cholesterol and apolipoprotein values, unit changes are expressed as mg/dL.
